# Opportunities to Retrofit, Reform, and Reimagine Systematic Reviews for Racial Equity

**DOI:** 10.1089/heq.2023.0150

**Published:** 2024-08-26

**Authors:** Darchelle V. Excellent, Bonnie Jones-Hepler, Amelia N. Gibson, Megan von Isenburg, Kristin P. Tully, Debra H. Brandon

**Affiliations:** ^1^ Duke University School of Nursing, PhD Program, Durham, North Carolina, USA.; ^2^ Duke University School of Nursing, Durham, North Carolina, USA.; ^3^ Family Connects International, Durham, North Carolina, USA.; ^4^ College of Information Studies, University of Maryland at College Park, College Park, Maryland, USA.; ^5^ Duke University Medical Center Library, Durham, North Carolina, USA.; ^6^ University of North Carolina at Chapel Hill, Department of Obstetrics and Gynecology, Chapel Hill, North Carolina, USA.; ^7^ Duke University Medical Center Library, Durham, North Carolina, USA.

**Keywords:** systematic review, racism, literature, controlled vocabulary, nursing science, power

## Abstract

Systematic reviews are used for synthesizing and summarizing published research on any given topic and population of interest. These reviews can expand knowledge within a content area but are limited by place and time values, which can perpetuate bias and systemic racism. This article reports a student's experience with conducting a systematic review on breastfeeding experiences among black birthing parents. We explore the systematic racism perpetuated by the current systematic review search process. We then use McLemore's “Retrofit, Reform, and Reimagine” framework for health equity to propose ways to increase transparency and racial equity through the systematic review process and academic mentoring.

## Introduction

Black scholars navigate white supremacy throughout their careers, contending with frustratingly durable systems of knowledge that subtly reproduce and concretize racist epistemologies in ways that are not always perceivable by peers, colleagues, and mentors.^1^ Reflecting on and sharing experiences can inform strategies to improve interdisciplinary research practices and help reform these systems.

This firsthand account offers insight into harm from the perspective of a black junior scholar engaging in the systematic review process for the first time. Even though researchers should strive to conduct all systematic reviews with health equity in mind, our experience was within the clinical setting, and we will focus on this specific setting. However, this commentary offers the teams' thoughts and recommendations as opportunities to strengthen the wider research community in other nonclinical settings as well.

Critical scholars within librarianship and the information sciences have long recognized metadata's role in reflecting and shaping researchers' social, political, and scientific understandings of the world.^2^ For the trainees in the health sciences, this is particularly salient, as systematic reviews, which are widely considered essential for establishing evidence-based practice,^3^ rely heavily on the complex frameworks constructed by descriptive subject headings, keywords, and catalog numbers. In this way, medical librarianship is critical in “the articulation and socialization of racial meanings”^4^ that shape clinicians' and researchers' worldviews.

The debate around racism in medical subject headings has resurfaced repeatedly since 2020, with many decrying the addition of “blacks” as a descriptor for black and African American people to MESH in 2022.^5^ In an open letter to NIH, a group of librarians and PubMed users asked for greater transparency in MESH term creation, demanding that the agency reduce harm by recognizing, “the problematic nature of outdated and offensive terms, and take into account the identifiers used by the communities themselves.”^5^

That year, the first author confronted this harm firsthand while working with a team of librarians to develop queries for an unpublished systematic review describing the breastfeeding experiences of black postpartum birthing parents in the United States. To build a complete data set related to black people, they would need to construct a list that reflected racism that was contemporaneous with the medical and nursing literature published within the search period. Discomfort with clarity around racial constructs created a system of evolving “colorblind” but racist proxies for race over time.

Rather than simply targeting population by race using terms describing race (e.g., black, African American, or Negro), the query would need to include stereotypical associations related to class and geography (e.g., “poverty,” “low income,” “inner city,” “urban,” and “slums) or risk excluding relevant publications. These associations would not be necessary for a search related to white birthing people. This study to advance the well-being of *all* black birthing people during their postpartum hospitalization (regardless of income, class, education level, or other proxy) would instead be built on perpetuation of deficit-based stereotypes. As Bowleg^6^ writes, “The language that researchers use shapes virtually every aspect of the research process.”

Clear and intentional instruction that acknowledges these limitations can help mitigate some of the harm here—especially for black researchers who might find the process personally traumatic. Junior scholars and their mentors (who might share racial or ethnic concordance) might have few opportunities and little meaningful support for critical engagement with complex bibliometric systems. Librarians' role is typically to contribute systematic review rigor to ensure scientific comprehensiveness, but they are not always prepared to deal with racial trauma or conversant in cultural considerations.

Fortunately, the authors of this article were able to support the lead author through honest discussions about intentional selection of key words and improved clarity. Other students or faculty might not be equipped to engage in this type of critical interrogation. Taking a reactive (rather than a proactive) approach to resolving these issues also disadvantages marginalized students who might feel disempowered or uncomfortable starting a potentially intractable conversation about racism with reluctant authority figures.

We are morally obligated to confront our publication and teaching practices if we hope to advance justice through research. This is especially relevant for researchers in the health care sector because one of the basic foundations of health care is to first do no harm. Our perspectives directly impact the ways we engage in knowledge generation and how we practice. We applaud the ongoing work by librarians dedicated to improving this problem and suggest a few interventions for more supportive and productive collaboration around systematic reviews. We structure our suggestions within McLemore's framework of “Retrofit, Reform, and Reimagine” for health equity to offer best practices to strengthen systematic reviews and academic mentoring.

## Retrofit

As McLemore outlines, we can retrofit gaps in scientific approaches by implementing workarounds and adopting individual-level interventions in the context of current, sometimes oppressive systems. This starts first by recognizing and acknowledging the historical and current context for research with marginalized communities. The systematic review process is multifaceted and, therefore, necessitates that we are vocal and clear about the racism embedded in our controlled vocabularies so that we can provide mentoring that is aware, empathetic, and responsive for researchers (particularly black and African American researchers) who work with them on a regular basis.

Only then can mentees develop positive strategies to navigate the current system. In this case, after discussing this issue of key word selection with the mentoring team and the librarians, the student better understood the context of why these search terms were being used.

Mentors and senior colleagues, especially those racially nonconcordant with their junior counterparts, should be educated about the underlying racism associated with the systematic search process and help their junior counterparts to understand how it impacts their analysis of the search results. Researchers should normalize use of antiracist language, and community preferred language within their articles, and should be critical of “race neutral” or “colorblind” methodology and language that perpetuates the use of euphemisms rather than actual racial terms.

Although striving to better equip and support learners, we can also center the strengths of populations by including search terms that address positive aspects such as community and faith. Increased cultural sensitivity about the research process will ideally foster more active support for scholars of color involved in completing systematic reviews.

## Reform

McLemore describes reform as improving systems' structure to create more accommodating conditions to achieve health equity.^7^ Systematic reviews have been used to identify all relevant literature around a specific topic, synthesize the findings from clinical trials, and to advance evidence-based medicine.^8,9^ This study needs to continue, but in practice can be reformed to assign greater value to more diverse research approaches, strategies, and methodologies.^8,9^ Systematic review methodology for specific scientific methods is outlined by the Preferred Reporting Items for Systematic Reviews and Meta-Analyses guidelines and the *Cochrane Handbook for Systematic Reviews of Interventions*, or other methodological guidance.^10,11^

Development and testing of comprehensive searches on complex topics take a great deal of time and expertise, and search terms are often created in consultation with librarians. Greater attention should be paid to the dynamics of this interprofessional relationship, so that students understand how and when librarians can help them think through these challenges.

Because of euphemisms in research articles, the slow-changing nature of controlled vocabularies, and the use of pre-existing search strategies, development of comprehensive searches for systematic reviews is primed for perpetuating harm and racism. Outdated linguistic and substantive standards for equity and antiracism are often not considered acceptable reasons for exclusion or points of evaluation for systematic reviews, respectively. The evolution of review methodology should address these issues.

## Reimagine

The academic system is primed for perpetuating racism,^8,9^ but can be changed. To reimagine is to dream and work to make those visions real. We reimagine that future bodies of evidence will promote equity and be antiracist. This might be achieved by leveraging artificial intelligence that can be regularly updated to crosswalk current vocabularies to (hidden) outdated (legacy) search terms; thus, preventing triggering exposures and reinforcement of biased associations. This could also be achieved by journals requiring that more inclusive and antiracist language are used in publications. Critical appraisal can give rise to new norms in primary research where more diverse sample selection and design should be considered.

In addition, all publications should consider more accurate and strength-based vocabularies when describing racial and ethnic populations. Antiracist leaders who understand how systematic racism influences library systems could be involved in regularly updating terminology related to black people and other misrepresented groups. As society changes, so will language. Foregrounding diverse expertise—in this case, that of a black doctoral student—supports more productive and generative collaborations among librarians, researchers, and the communities we partner with and strive to serve.^5,12^

## A Call to Action

The process of retrofitting, reforming, and reimagining is not a linear process, but rather cyclical.^7^ It is constant and takes time and effort. A “checklist approach” to achieve health equity will not solve the problem in systematic reviews nor in other domains. Health equity is not an end-point, but rather an active continual collaboration for people to rebuild systems to optimize wellness. Thus, re-evaluation and interventions must be ongoing because of the ever-changing landscape of language.

## Conclusion

This article highlights components of racism in academia through reflection of the systematic review search process. We offer ways to retrofit, reform, and reimagine the systems to where it can actively become antiracist and more enjoyable. Although this experience is from systematic reviews conducted in the clinical setting, this experience can be generalized to all systematic reviews. As researchers, librarians, and students, we all have a role to play in confronting and dismantling systematic racism in all settings.

Acknowledgment of these issues and speaking up to initiate the conversations about the change that needs to happen should be encouraged and supported. Our silence to systems that perpetuate racism is deafening and ignores one of the basic foundations of health care, which is to first do no harm.

## References

[1] Jones S, Flowers A, Palmer R. Black Scholarship in a White Academy: Perseverance in the Face of Injustice. Johns Hopkins University Press CY: Baltimore, MD, USA; 2023.

[2] Olson HA. The power to name: Representation in library catalogs. Signs 2001;26(3):639–668.

[3] Holly C, Salmond S, Saimbert M. Systematic Review as a Basis for Evidence-Based Practice. Springer Publishing Company: New York, NY, USA; 2017; pp. 1–16.

[4] Honma T. Trippin’ over the color line: The invisibility of race in library and information studies. Interact UCLA J Educ Inform Stud 2005;1(2); doi: 10.5070/D412000540

[5] Roth S. Open Letter to NLM Regarding MeSH Term Changes. 2022. Available from: https://www.mlanet.org/p/cm/ld/fid=1122&&blogaid=4008 [Last accessed: April 1, 2024].

[6] Bowleg L. “The master's tools will never dismantle the master's house”: Ten critical lessons for black and other health equity researchers of color. Health Educ Behav 2021;48(3):237–249; doi: 10.1177/10901981211007402 34080476

[7] McLemore MR. Using retrofit, reform, and reimagine to advance toward health equity. J Perinat Neonatal Nurs 2022;36(2). 10.1097/JPN.0000000000000639 35476759

[8] Clarke M, Chalmers I. Reflections on the history of systematic reviews. BMJ Evid Based Med 2018;23(4):121–122; doi: 10.1136/bmjebm-2018-110968 29921711

[9] Clarke M. History of evidence synthesis to assess treatment effects: Personal reflections on something that is very much alive. J R Soc Med 2016;109(4):154–163; doi: 10.1177/0141076816640243 27059906 PMC4827109

[10] Page MJ, McKenzie JE, Bossuyt PM, et al. The PRISMA 2020 statement: An updated guideline for reporting systematic reviews. BMJ 2021;372:n71; doi: 10.1136/bmj.n71 33782057 PMC8005924

[11] Cochrane Handbook for Systematic Reviews of Interventions. n.d. Available from: https://training.cochrane.org/handbook [Last accessed: April 1, 2024].

[12] Townsend W, Anders P, Capellari E, et al. Addressing antiquated, non-standard, exclusionary, and potentially offensive terms in evidence syntheses and systematic searches. 2022. Available from: https://deepblue.lib.umich.edu/handle/2027.42/174677 [Last accessed: April 1, 2024].

